# Insights and Constraints: Understanding the Boundaries in Investigating Otosclerosis and Endolymphatic Hydrops Interaction

**DOI:** 10.7759/cureus.99962

**Published:** 2025-12-23

**Authors:** Melissa Castillo-Bustamante, Carlos Guajardo-Vergara, Enrico Armato

**Affiliations:** 1 Otolaryngology, Clinica Universitaria Bolivariana, Medellín, COL; 2 Otoneurology, Centro de Vértigo y Mareo, Medellín, COL; 3 School of Medicine, Health Sciences School, Universidad Pontificia Bolivariana, Medellín, COL; 4 Audiology, Escuela de Fonoaudiología, Universidad Austral de Chile, Puerto Montt, CHL; 5 Neurosciences Department, University of Padova, Padova, ITA

**Keywords:** anatomy & physiology, otosclerosis, peripheral vertigo, vestibular assessment, vestibular disorders

## Abstract

Otosclerosis and endolymphatic hydrops (EH) are distinct inner ear pathologies that may coexist within the same temporal bone, creating diagnostic and therapeutic challenges. Their interaction remains controversial, with proposed mechanisms suggesting that otosclerotic involvement of the vestibular aqueduct or cochlear endosteum may obstruct endolymphatic flow, leading to secondary hydrops. This review summarizes current evidence on the coexistence, pathophysiology, diagnostic tools, and clinical implications of otosclerosis-associated EH.

Histopathologic studies have demonstrated EH adjacent to otosclerotic foci involving the cochlear endosteum and the vestibular aqueduct, supporting a mechanical or metabolic interaction between both entities. Advances in magnetic resonance imaging, particularly gadolinium-enhanced 3D-fluid-attenuated inversion recovery (FLAIR) sequences, have allowed in vivo visualization of cochlear and vestibular hydrops, improving diagnostic accuracy and enabling earlier detection of mixed pathology. Clinically, affected patients often exhibit mixed or fluctuating hearing loss, tinnitus, and vertigo, suggesting both cochlear and vestibular compromise. Post-stapedectomy EH has also been described, likely secondary to altered perilymphatic pressure or microtrauma.

Despite growing evidence supporting an association, the causal relationship remains uncertain - whether EH arises as a secondary consequence of otosclerosis or represents a concurrent process within a shared pathophysiological spectrum. The coexistence of EH and otosclerosis exemplifies the complexity of inner ear disorders, highlighting the need for integrated approaches combining imaging, audiovestibular testing, and histopathologic correlation. Multidisciplinary collaboration and longitudinal studies are essential to clarify underlying mechanisms, refine diagnostic criteria, and optimize management strategies for patients with combined otosclerotic and hydropic pathology.

## Introduction and background

The intricate labyrinth of the inner ear harbors a myriad of challenges for clinicians and researchers alike [1]. Among the intriguing phenomena encountered within this delicate auditory domain, the simultaneous presence of endolymphatic hydrops (EH) and otosclerosis has emerged as a captivating area of exploration [1]. As two distinct pathologies, each posing unique implications for auditory and vestibular function, the convergence of hydrops and otosclerosis within the temporal bone raises interesting questions about their interplay and shared mechanisms [1].

The simultaneous presence of otosclerosis and EH in the temporal bone has been documented; nonetheless, the precise mechanism underlying the development of EH in otosclerosis remains unclear [2]. Some studies have suggested that the obstruction caused by otosclerotic changes in the vestibular aqueduct might disrupt the outflow or absorption of endolymph, potentially resulting in the development of EH [2]. Likewise, EH may coexist with otosclerosis preoperatively, suggesting two separate concurrent diseases, or EH might be induced by the otosclerotic process itself [1]. Additionally, EH can occur postoperatively as a result of a fistula, particularly following stapedectomy. In such cases, surgery to seal the fistula effectively treats EH, leading to a subsiding of symptoms and an improvement in hearing [1]. Clinical manifestations of EH associated with otosclerosis include conductive or mixed-type hearing loss in the initial stages, a feeling of fullness in the affected ear, tinnitus, fluctuating hearing, episodes of vertigo, and changes in the electrocochleography [3].

The relationship between otosclerosis and EH has been explored through clinical and radiologic studies, animal models, and further examinations of human otopathology [4]. Otosclerosis is a causative factor in the development of the EH [4]. EH is linked to otosclerotic lesions that either reach the cochlear endosteum or involve the vestibular aqueduct, obstructing the longitudinal flow of endolymph. Additionally, surgical interventions for otosclerosis may lead to immediate or delayed iatrogenic EH [5]. 

In elucidating the literature surrounding otosclerosis and EH, the intention is to synthesize current research, highlight significant advancements, and underscore persistent challenges in the diagnosis and management of these disorders [5]. Additionally, this effort aims to elucidate the principal histopathological changes and delineate the pathophysiological relationships correlating with these alterations, thereby contributing to a comprehensive understanding of the disease spectrum.

## Review

Methods

This study was designed as a narrative review to comprehensively map and characterize the existing literature on the relationship between otosclerosis and EH. The review was conducted and reported in accordance with the Preferred Reporting Items for Systematic reviews and Meta-Analyses (PRISMA) guidelines.

Search Strategy

A systematic literature search was performed across PubMed/MEDLINE, Scopus, and Web of Science from January 1969 through December 31, 2024. The search strategy combined controlled vocabulary terms (when applicable) and free-text keywords related to otosclerosis and hydrops, including but not limited to: “otosclerosis,” “endolymphatic hydrops,” “cochlear hydrops,” “vestibular hydrops,” “otosclerosis and vestibular disorders,” and “otosclerosis and vestibular testing.” Reference lists of included articles were also manually screened to identify additional relevant studies.

Eligibility Criteria

Eligibility criteria were defined using a Population-Concept-Context framework. We included studies involving adult patients (≥18 years) with otosclerosis in whom endolymphatic or cochlear hydrops was assessed clinically, radiologically, histopathologically, or physiologically. The concept of interest was the presence, characterization, or clinical implications of hydrops in the context of otosclerosis.

Eligible study designs included case reports, case series, case-control studies, observational cohorts, imaging studies, and histopathological analyses. Articles published in English or in other languages with available translations were considered. We excluded pediatric studies, as otosclerosis and hydrops in children represent distinct etiological and clinical entities. We also excluded conference abstracts, non-peer-reviewed publications, systematic reviews, and meta-analyses, as the objective of this narrative review was to map primary evidence.

Study Selection

**Figure 1:**
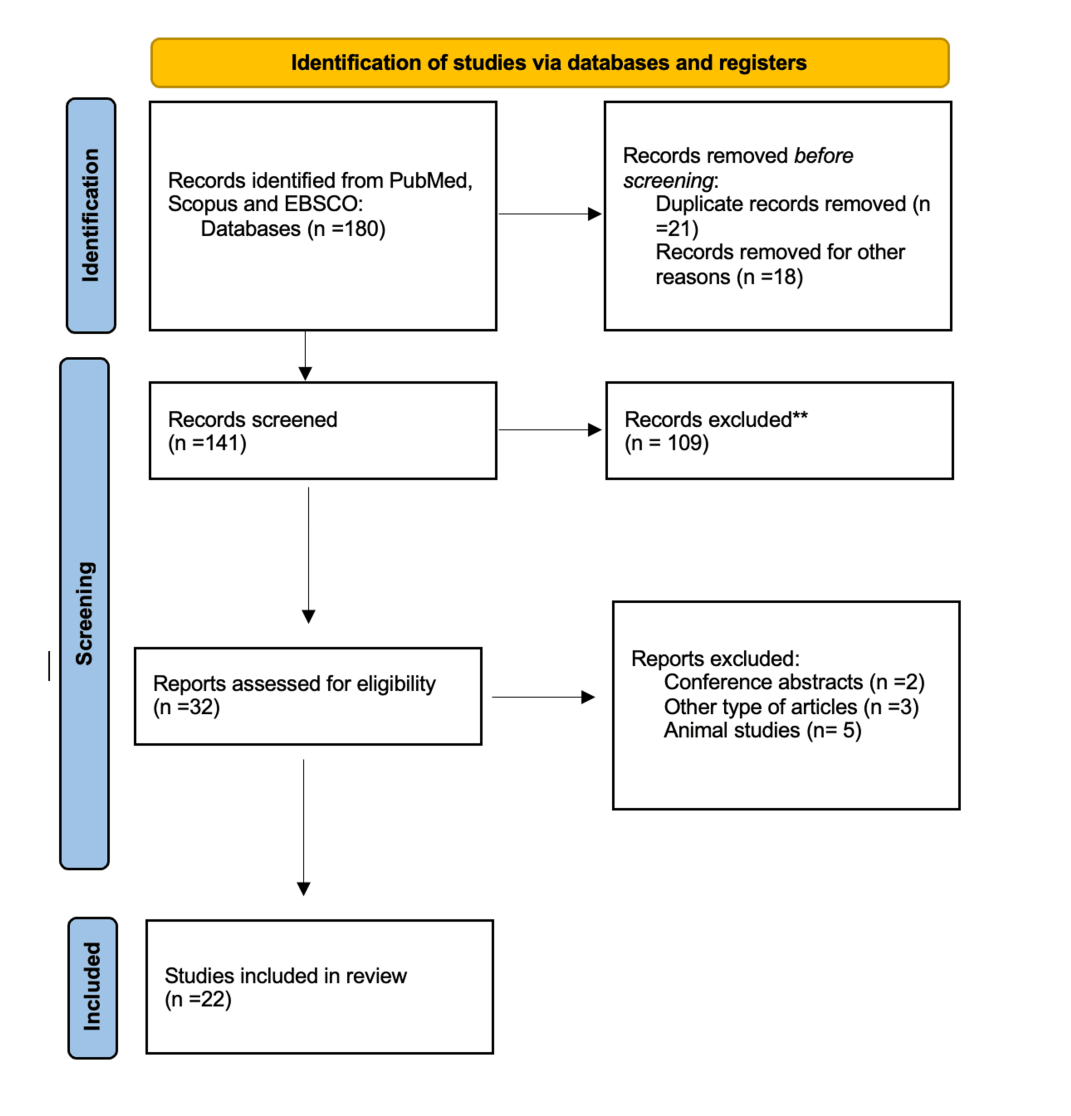
Flow diagram of the literature search and study selection process.

Study selection was conducted in a two-stage process. First, titles and abstracts were independently screened for relevance. Second, full-text articles of potentially eligible studies were assessed for final inclusion. Discrepancies were resolved through discussion and consensus. The study selection process and reasons for exclusion are summarized in a PRISMA flow diagram (Figure 1).

Data Extraction

Data were extracted using a standardized data collection form developed a priori. Extracted variables included study design, sample size, patient characteristics, diagnostic criteria for otosclerosis and hydrops, imaging or histopathological methods, vestibular or audiological findings, and reported clinical outcomes. Data extraction was performed with cross-checking to ensure accuracy and consistency.

Quality Considerations and Synthesis

Given the heterogeneity of study designs and the exploratory aim of this review, no quantitative meta-analysis was performed. Findings were synthesized narratively, with descriptive summaries of study characteristics and reported outcomes. Although formal risk-of-bias assessment is not mandatory for narrative reviews, we explicitly considered methodological limitations and potential sources of bias across studies, particularly those related to small sample sizes, retrospective designs, and selective reporting.

Bias and Limitations

Potential publication and language biases are acknowledged, as gray literature and non-peer-reviewed sources were excluded, and only studies with accessible translations were included. These factors may favor the reporting of positive findings and are considered when interpreting the results.

Facts in Clinics and Pathophysiology

**Figure 2:**
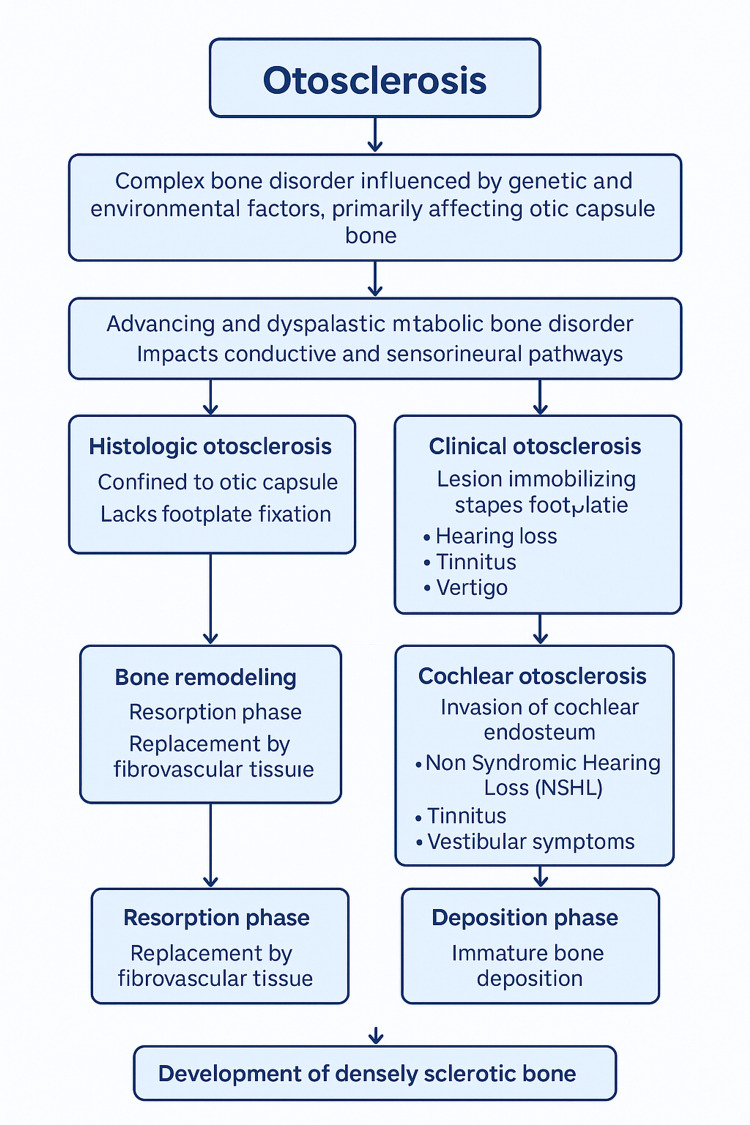
Main histopathological and clinical findings in otosclerosis Schematic representation created by the authors for this manuscript. This figure is original, has not been previously published, and was not created from external sources. The authors grant permission for its reproduction.

Otosclerosis is a complex bone disorder influenced by both genetic and environmental factors, primarily affecting the otic capsule bone [5]. It is an advancing and dysplastic metabolic bone disorder exclusive to humans, impacting both the conductive and sensorineural pathways by inducing alterations in the middle and inner ear [5]. This condition leads to a gradual onset of conductive hearing loss, primarily attributed to the fixation of the stapes footplate [6]. Nevertheless, in 10% of otosclerosis cases, there is a sensorineural component leading to either mixed hearing loss or, rarely, a pure sensorineural hearing loss (SNHL) [6,7]. It stands as a prevalent contributor to acquired hearing loss [7]. This disorder can be categorized based on either clinical presentation or histopathologic findings [8]. Histologic otosclerosis specifically pertains to cases confined to the otic capsule and lacks footplate fixation or discernible clinical consequences [8]. As such, it is typically an incidental discovery during temporal bone autopsies [8]. On the other hand, clinical otosclerosis is characterized by a lesion that immobilizes the stapes footplate, accompanied by auditory and vestibular symptoms such as hearing loss, tinnitus, and vertigo [8]. Additionally, cochlear otosclerosis involves the invasion of the cochlear endosteum, extensively affecting the otic capsule without stapes fixation [9,10]. This form of otosclerosis leads to non-syndromic hearing loss (NSHL), tinnitus, and vestibular symptoms [9,10]. Figure 2 illustrates the most relevant histopathological and clinical findings associated with otosclerosis.

Initially, bone experiences a resorption phase, during which it is replaced by cellular fibrovascular tissue surrounding blood vessels [11]. Subsequently, there is a continuous and simultaneous occurrence of immature and collagen-deficient bone deposition within a specific focus, initiating a cycle of continuous resorption and remodeling [11,12]. Over time, bone deposition takes place, accompanied by an increased concentration of collagen and a diminished ground substance [11]. This process ultimately leads to the development of densely sclerotic bone characterized by a prominent woven pattern of cement lines [11,12]. The distinctive pathological feature of otosclerosis involves anomalous remodeling of bone structure, encompassing processes such as bone resorption, the formation of new bone, and increased vascular proliferation within the temporal bone [6]. When observed under light microscopy, the active stage, characterized by the replacement of bone structure with new bone, can be distinguished from the inactive stage, during which the bone undergoes significant mineralization and thickening [6].

EH is a pathologic finding characterized by the distension of structures surrounding the endolymphatic space due to an increase in the volume of endolymph [13]. This condition is associated with Ménière's disease but is not limited to it; EH can result from various factors such as trauma, infection, obstruction, or unknown origins [13]. Its acute impact on hearing and balance stems from ruptures in Reissner’s membrane and alterations in the concentration of electrolytes in the endolymph [13]. Episodic vertigo and fluctuating hearing loss have been linked to the presence of EH [14]. This disorder is characterized by the abnormal buildup of endolymphatic fluid within the inner ear's membranous labyrinth and is underpinned by a complex pathophysiology [13]. The disruption of fluid homeostasis plays an important role in this condition, where the balance of endolymph production, circulation, and absorption within the inner ear is compromised [15]. The increased pressure affects the vestibular system, particularly the semicircular canals, which play a crucial role in balance and spatial orientation; disturbances in this system result in vertigo and a sense of imbalance [15].

While traditionally viewed as separate entities, emerging evidence suggests potential interplay between otosclerosis and EH, however, the link between these two entities continues to be a source of controversy [1]. Different mechanisms have been described where otosclerosis foci produce vertigo; among them, we find both direct neural degeneration and biochemical changes produced by the contact of perilymph with the foci, altering its biochemistry [1]. Otosclerosis is one of many factors associated with hydrops although it is more common than is usually reported [1,16]. Considering the impact of EH on the maculae of the saccule and utricle is essential for a comprehensive understanding of vestibular manifestations in Ménière’s disease and other secondary hydropic conditions [17]. Recent studies have demonstrated that involvement of these otolithic organs can contribute to symptoms such as chronic unsteadiness, persistent imbalance, or drop attacks, which can significantly affect patients’ quality of life even when episodes of rotational vertigo are infrequent or well controlled [17]. One study described that saccular and utricular distention can be visualized using delayed intravenous gadolinium-enhanced 3D-fluid-attenuated inversion recovery (FLAIR) MRI, providing direct evidence of otolithic involvement in the pathophysiology of EH and offering a non-invasive method to evaluate inner ear structures [17]. Additionally, Barlet et al. optimized 3D-FLAIR sequences to reduce the interval between gadolinium administration and image acquisition, facilitating faster and more reliable detection of EH in clinical practice [18]. These imaging advances not only enhance diagnostic confidence but also allow correlation of radiological findings with specific vestibular symptoms [18]. Recognition of saccular and utricular involvement in EH may help explain residual imbalance in patients who have otherwise responded well to treatment for vertigo attacks, underscoring the need for comprehensive evaluation beyond semicircular canal function alone [18]. Imaging confirmation of otolithic hydrops could inform individualized rehabilitation strategies, including vestibular therapy focusing on otolith-specific exercises aimed at improving balance and reducing fall risk [17,18]. Moreover, discussing otolithic involvement with patients may provide valuable insights into prognosis, help set realistic expectations, and guide lifestyle modifications [17,18]. Incorporating assessment of otolithic function into the routine evaluation of suspected hydropic ear disease could therefore improve diagnostic accuracy, support personalized treatment plans, and ultimately enhance patient outcomes [17,18]. The examination of temporal bones provides compelling insights into a potential interconnection between EH and otosclerosis [4]. The intricate relationship observed within these anatomical structures suggests a significant impact on the production and resorption of fluid [4]. Notably, in affected bones with more extensive involvement, there is a discernible increase in fluid volume, indicating a direct pathophysiological association that unveils a distinct spectrum of the disease [4]. Liston et al.'s study, evaluating 95 temporal bones with otosclerosis foci, unveiled cochlear hydrops in six instances, two of which demonstrated foci extending to the cochlear endosteum [4]. This extension affected the position of the cochlear membrane, specifically the Reissner membrane, contributing to the pathogenesis of EH [4]. The manifestation of EH in otosclerosis is linked to alterations in intracochlear ionic balance and obstruction of the endolymphatic duct and sac when the spiral ligament is affected [7]. Interestingly, the presence and severity of EH in patients with otosclerosis do not necessarily align with the intensity of symptoms [1,13]. These findings underscore the intricate relationship between otosclerosis and EH, shedding light on the nuanced pathophysiological mechanisms governing their coexistence [1,13].

Clinical and Imaging Diagnosis

The audiometric findings in individuals with otosclerosis and EH can vary, presenting a combination of conductive, sensorineural, or mixed hearing loss. Otosclerosis often leads to conductive hearing loss due to the fixation of the stapes footplate, restricting its movement in the oval window [14]. EH, particularly in the cochlea, can contribute to sensorineural hearing loss due to changes in fluid dynamics within the inner ear [1,13]. Mixed hearing loss, a combination of conductive and sensorineural components, reflects the simultaneous impact of otosclerosis and hydrops on hearing [9]. Hydrops, characterized by fluid imbalances, may cause fluctuating hearing loss and vertigo with episodes of improvement and worsening [9]. The diagnosis of EH concomitant with otosclerosis presents a multifaceted challenge that requires a comprehensive approach [19]. Audiological assessments, including pure-tone audiometry and speech audiometry, play a crucial role in evaluating the impact of otosclerosis on hearing function [19]. Complementary imaging modalities, such as high-resolution temporal bone computed tomography (CT) scans, aid in identifying otosclerotic foci and assessing the extent of their involvement [20]. Additionally, advanced imaging techniques like magnetic resonance imaging (MRI) contribute valuable information about soft tissue changes and potential manifestations of EH [21]. Vestibular function assessments, such as electronystagmography (ENG) or videonystagmography (VNG), are instrumental in detecting vestibular symptoms associated with hydrops, providing a more comprehensive diagnostic picture [8]. In some cases, diagnostic tools specific to EH, such as electrocochleography (ECochG) or vestibular evoked myogenic potentials (VEMP), may be employed to capture subtle changes [22]. ECochG typically reveals an elevated negative summating potential (SP) and an increased ratio of summating potential and action potential (SP/AP), which are characteristic findings in EH [23]. Similarly, VEMP testing reveals changes in wave amplitude due to tuning effects at frequencies of 0.5 and 1 kHz, which are particularly sensitive indicators of EH [23]. These tuning effect changes, reflected in wave amplitude modifications at these specific frequencies, are attributed to the increased endolymphatic volumetry, a finding that has been corroborated through magnetic resonance imaging studies in hydrops patients [23]. 

Recent evidence suggests that there is a significant association between otosclerosis and the development of secondary EH, which can manifest as symptoms resembling Ménière’s syndrome [24]. Rajan et al. highlighted that cochlear otosclerosis can lead to secondary EH due to abnormal bone remodeling around the otic capsule, potentially disturbing inner ear fluid homeostasis and causing fluctuating sensorineural hearing loss, vertigo, or both [24,25]. One study analyzed clinical features of patients with both otosclerosis and EH, demonstrating that these patients often present with atypical patterns of hearing loss and vestibular symptoms, suggesting that concurrent EH may complicate the clinical course of otosclerosis and contribute to persistent imbalance or vertigo [2]. Furthermore, Ishai et al. evaluated the incidence of EH following stapedectomy in patients with otosclerosis, reporting that 7.3% of patients developed low-frequency sensorineural hearing loss (LFSNHL) after surgery, despite successful improvement in conductive hearing loss [25,26]. Histopathological analysis further revealed a higher incidence of EH in temporal bone specimens (TBS) from operated patients (11.8%) compared to non-operated otosclerotic TBS (1.9%) and control TBS from individuals with presbycusis (3.5%), demonstrating a statistically significant association between stapedectomy and the presence of EH (p < 0.001) [26]. Collectively, these findings emphasize the importance of considering secondary EH as a potential cause of persistent or delayed-onset vestibular complaints in otosclerosis patients, both before and after surgical intervention [26]. Identifying EH through imaging or detailed vestibular evaluation could aid in tailoring postoperative management and counseling patients regarding possible vestibular sequelae [25,26].

Recent imaging studies have highlighted a potentially complex relationship among EH, otosclerosis, and superior semicircular canal dehiscence (SSCD), which may coexist and contribute to overlapping auditory and vestibular symptoms [27]. EH was identified on MRI in 27.3% of ears with SCDS (nine out of 33 ears); however, no significant correlation was found between the presence of EH on MRI and cervical or ocular VEMPs (cVEMP or oVEMP), which are considered the gold standard tests in these cases [27]. Given that otosclerosis can also disturb inner ear fluid homeostasis, the simultaneous presence of otosclerosis, EH, and SSCD may create a multifactorial substrate for fluctuating hearing loss, sound- or pressure-induced vertigo, and imbalance [26]. Recognizing this interplay is crucial, as it can affect diagnostic accuracy and influence surgical decision-making, particularly in cases where symptoms persist despite treating one of these conditions in isolation [26]. Comprehensive imaging and vestibular testing should therefore be considered in patients with complex or refractory symptoms suggestive of multiple coexisting inner ear pathologies [26].

Challenges and Controversies

The intersection of EH and otosclerosis introduces a set of challenges and controversies that contribute to the ongoing discourse within the audiological and otological communities [1]. One prominent challenge lies in the diagnostic realm, where distinguishing between the distinct pathologies of EH and otosclerosis poses difficulties due to overlapping symptoms and shared clinical manifestations [1]. This diagnostic ambiguity often necessitates a comprehensive and multidisciplinary approach, involving audiologists, radiologists, and otologists, to unravel the intricacies of these coexisting conditions. Controversies arise regarding the exact mechanistic relationship between hydrops and otosclerosis [1,28]. While some studies suggest a potential direct pathophysiological connection, others emphasize the coexistence without a causal relationship [1,27]. Clarifying the nature of this association remains a focal point of debate within the scientific community, impacting the development of targeted therapeutic strategies [1,27].

The varying degrees of symptomatology further complicate the clinical landscape. EH and otosclerosis can manifest with a wide spectrum of symptoms, ranging from subtle auditory changes to debilitating vertigo [28]. Deciphering the clinical significance of these symptoms and their correlation with disease progression is an ongoing challenge, particularly given the intricate nature of the auditory system and the individual variability in patient responses [29]. Treatment strategies also encounter challenges, with controversies surrounding the optimal management of individuals presenting with both EH and otosclerosis [1,29]. Balancing interventions to address each condition while minimizing potential adverse interactions remains an area of active research and debate [1,29]. The lack of standardized guidelines for such cases adds an additional layer of complexity to clinical decision-making [1,29]. Moreover, the long-term outcomes and prognosis of individuals with concurrent EH and otosclerosis remain uncertain. Predicting disease progression and understanding the impact on overall quality of life constitute areas of ongoing investigation, prompting discussions on the need for longitudinal studies to provide more comprehensive insights [1,29].

This comprehensive review of literature, utilizing a narrative approach, sought to synthesize current research, highlight advancements, and underscore persistent challenges in the diagnosis and management of these disorders [1,29]. Histopathological changes, particularly the involvement of the cochlear endosteum and the vestibular aqueduct, contribute to the pathogenesis of EH in otosclerosis [1,29]. However, the precise mechanisms underlying the development of EH in otosclerosis remain unclear, with debates over whether EH is a result of separate concurrent diseases or induced by the otosclerotic process itself [1,29]. The diagnostic landscape is marked by multifaceted challenges, including overlapping symptoms and shared clinical manifestations [28,29]. Distinguishing between otosclerosis and EH requires a nuanced, multidisciplinary approach, involving audiologists, radiologists, and otologists [29]. Controversies surrounding the mechanistic relationship impact the development of targeted therapeutic strategies, necessitating further research to elucidate causal pathways [29].

Several limitations should be considered in interpreting the findings of this comprehensive review. The study primarily focused on literature available up to 2024, potentially overlooking more recent developments in the field. The inclusion of articles written only in English and those with available translations may introduce a language bias, limiting the global representation of research on otosclerosis and EH. The reliance on peer-reviewed articles might introduce publication bias, as positive findings are more likely to be published, neglecting unpublished studies or negative results. The diverse study designs, including clinical studies, case-control studies, and case reports, introduce heterogeneity in quality and methodology, impacting the overall robustness of conclusions. The inherent diagnostic challenges associated with otosclerosis and EH, especially when coexisting, contribute to variability in reported findings. The lack of standardized diagnostic criteria, treatment protocols, and outcome measures across studies further complicates result synthesis. Additionally, potential bias in the selection process and the exclusion of conference abstracts and systematic reviews may influence the diversity of perspectives considered. Lastly, the generalizability of findings may be limited, as most studies may have focused on specific populations or clinical scenarios. These limitations underscore the need for cautious interpretation and highlight areas for future research to address these gaps in knowledge and methodology.

## Conclusions

In conclusion, the coexistence of otosclerosis and EH presents a challenging clinical scenario. While there is evidence suggesting a potential relationship between these conditions, the exact mechanisms remain debated. Diagnostic challenges stemming from overlapping symptoms emphasize the need for a multidisciplinary approach. Controversies surrounding causality impact treatment strategies. Long-term outcomes and quality of life in affected individuals require further investigation. This review provides valuable insights, but limitations highlight the need for continued research to better understand and manage this complex interaction between otosclerosis and EH.
